# New Scale of Salaries at the London Hospital

**Published:** 1919-04-05

**Authors:** 


### NEW SCALE OF SALARIES AT THE LONDON HOSPITAL.

### Increase of £7,259 a Year.

BEFORE Miss Luckes died the question of revising salaries at the London Hospital had been under much discussion, and Lord Knutsford alluded to the coming changes in his speech at the quarterly court of governors on March 5. In the scheme now approved no fewer than 672 sisters and nurses are affected, and the increase, which amounts this year to £7,259, will, under the progressive scale, be very considerably extended as time goes on. In order to present a clear idea of this generous and remarkable scheme the staff has been divided into seven groups. But there are certain conditions of service common to the whole staff which considerably augment the value of the salaries.

### CONDITIONS OF SERVICE.

Full board and lodging, with separate bedroom for every member of the staff, is estimated on its actual cost to the hospital at an average of £78 a year; laundry allowance is now raised to 3s. 6d. a week; dress material for uniform is given. A month's annual holiday is given on full pay and sick leave on full pay is indefinitely extended where there is hope of complete recovery. Under an unique arrangement all nurses have £5 added to their salary on completion of six years' service from date of entry as probationer, and a second £5 is added after twelve years' service. These grants to encourage long service are retained under the new scale. Besides this, £5 is added to the salary of any nurse or sister who has gained the C.M.B. certificate at her own expense. Finally a pension of full pay for life, based on the average salary for the last five years, and including the above additions is paid to all sisters and nurses who have served eighteen years and have reached the age of forty-five. Pensioners are at liberty to take other appointments. The age limit has now been removed, and the qualifying length of service increased to twenty years instead of eighteen.

### WHAT THE INCREASES PROVIDE.

*Group* 1, *Matron's Assistants.*—The seven persons in this group include besides the matron's assistants, the Sister-in-Charge of Tredegar House, and the Matron of the Convalescent Home at Felixstowe. Commencing at £60 a year their salaries rise under the new scale £10 a year, till the maximum of £150 a year is reached. Most of this group are now receiving their maximum at £100, and will be raised at once to £150, in addition to the long service and midwifery grant above mentioned. The pension for this group is fixed at £110, plus £5 extra for midwifery.

*Group* 2, *Heads of Departments.*—There are seven in this group, viz., the sister tutor, whose appointment was agreed to by the committee on March 24, and the heads of the private nursing department, the laundry, the massage department, the linen room, the nurses' home, and the Finsen light department. Commencing at £65 their salaries will rise £5 per annum until the maximum is reached of £110. At present these ladies are receiving from £60 to £75, and some of them will receive the maximum at once. The pension for this group will be £80 a year, plus midwifery addition.

*Group* 3, *Special Appointments.*—Under these posts, which are not quite equal in importance to the above, come night sisters and sisters in the following departments: Massage, receiving room, out-patients (medical and surgical), Marie Céleste and Victor wards, maternity district, matron's office, sick-room cookery, and light department (below the head). The salaries are to run from £55 to £100, reached in ten years by annual £5 increases, with service and midwifery additions besides. Night sisters who never hold office more than three years are to have special consideration, and are to receive £90, £95, and £100 in their years of office. Each year of night duty is to count for two in this group on account of the long and trying hours. Sisters Marie Céleste and Victor are to have £10 a year extra on account of their very special and extra duties in training midwifery pupils and students.

*Group* 4, *Ward Sisters and Holiday Sisters.*—There are about fifty-six sisters in this group at present earning from £40 to £50 a year. Under the new scale they begin at £50 and rise by yearly increments of £5 to £95. The pension obtainable by members of this group will be £60 a year, plus midwifery addition.

*Group* 5, *Private Staff Nurses.*—There are about 210 nurses at present in this group, of whom 87 are now earning £50, 15 are earning £45, 38 are earning £40, 70 are earning £35. Under the new scale they will commence at £45, and by yearly increase of £5 reach the maximum of £70 in six years, with service and midwifery additions extra. Their pension is to be £60, plus £5 for midwifery if acquired.

*Group* 6, *Ward Staff Nurses.*—About 132 of these nurses are now paid at the rate of £30 a year. These are third year nurses, and hold the post for one year only. They are now to have £40 a year.

**London Hospital Salaries—(*continued*).**

*Group* 7, *Probationers*.—There are 124 in their first year and 117 in their second. Salaries are to be raised from £17, £24, to £20, £25. We are heartily in agreement with the policy of spending money on raising the salaries of the sisters rather than those of the probationers, and would point out that the London Hospital already pays more to its probationers than most other establishments.

The scheme as it stands is in all respects worthy of the great institution from which it issues. It marks the attainment of a definite stage in nursing progress. Hitherto the administrative posts in hospitals have been grasped at, though poor in value, because they might lead to something better. The nurse put her best years into a lottery, as it were, and often enough drew a blank. From now on all this is changed. Hospitals which need to retain women of exceptional ability in their service are coming one after another, as our columns show this week, to recognise that they must pay the price. The London Hospital has set a fine example in this direction.

